# Non-Invasive Skin Cancer Diagnosis Using Hyperspectral Imaging for In-Situ Clinical Support

**DOI:** 10.3390/jcm9061662

**Published:** 2020-06-01

**Authors:** Raquel Leon, Beatriz Martinez-Vega, Himar Fabelo, Samuel Ortega, Veronica Melian, Irene Castaño, Gregorio Carretero, Pablo Almeida, Aday Garcia, Eduardo Quevedo, Javier A. Hernandez, Bernardino Clavo, Gustavo M. Callico

**Affiliations:** 1 Institute for Applied Microelectronics (IUMA), University of Las Palmas de Gran Canaria (ULPGC), 35017 Las Palmas de Gran Canaria, Spain; bmartinez@iuma.ulpgc.es (B.M.-V.); hfabelo@iuma.ulpgc.es (H.F.); sortega@iuma.ulpgc.es (S.O.); vmelian@iuma.ulpgc.es (V.M.); equevedo@iuma.ulpgc.es (E.Q.); gustavo@iuma.ulpgc.es (G.M.C.); 2 Department of Dermatology, Hospital Universitario de Gran Canaria Doctor Negrín, Barranco de la Ballena s/n, 35010 Las Palmas de Gran Canaria, Spain; irenecastano@hotmail.com (I.C.); gcarher@gobiernodecanarias.org (G.C.); 3 Department of Dermatology, Complejo Hospitalario Universitario Insular-Materno Infantil, Avenida Maritima del Sur, s/n, 35016 Las Palmas de Gran Canaria, Spain; pjalmmar75@gmail.com (P.A.); jhersant@gobiernodecanarias.org (J.A.H.); 4 Department of Electromedicine, Complejo Hospitalario Universitario Insular-Materno Infantil, Avenida Maritima del Sur, s/n, 35016 Las Palmas de Gran Canaria, Spain; agarcia@iuma.ulpgc.es; 5 Research Unit, Hospital Universitario de Gran Canaria Doctor Negrín, Barranco de la Ballena s/n, 35010 Las Palmas de Gran Canaria, Spain; bernardinoclavo@gmail.com

**Keywords:** hyperspectral imaging, skin cancer, clinical diagnosis, biomedical optical imaging, medical diagnostic imaging

## Abstract

Skin cancer is one of the most common forms of cancer worldwide and its early detection its key to achieve an effective treatment of the lesion. Commonly, skin cancer diagnosis is based on dermatologist expertise and pathological assessment of biopsies. Although there are diagnosis aid systems based on morphological processing algorithms using conventional imaging, currently, these systems have reached their limit and are not able to outperform dermatologists. In this sense, hyperspectral (HS) imaging (HSI) arises as a new non-invasive technology able to facilitate the detection and classification of pigmented skin lesions (PSLs), employing the spectral properties of the captured sample within and beyond the human eye capabilities. This paper presents a research carried out to develop a dermatological acquisition system based on HSI, employing 125 spectral bands captured between 450 and 950 nm. A database composed of 76 HS PSL images from 61 patients was obtained and labeled and classified into benign and malignant classes. A processing framework is proposed for the automatic identification and classification of the PSL based on a combination of unsupervised and supervised algorithms. Sensitivity and specificity results of 87.5% and 100%, respectively, were obtained in the discrimination of malignant and benign PSLs. This preliminary study demonstrates, as a proof-of-concept, the potential of HSI technology to assist dermatologists in the discrimination of benign and malignant PSLs during clinical routine practice using a real-time and non-invasive hand-held device.

## 1. Introduction

Skin cancer is categorized as non-melanoma skin cancer (NMSC) and melanoma [1]. NMSC (excluding basal-cell carcinomas, BCCs) was the 5th most common form of cancer worldwide in 2018, involving over 1 million of new diagnoses and 65,000 death, while melanoma was the 21th with nearly 300,000 new cases and 60,000 death [1]. In pigmented skin lesions (PSLs), an extreme progression of melanocytes, which are pigment-producing cells in the basal layer of the epidermis, is found. PSLs can be classified as benign or malignant [2]. The most common NMSC are BCC and squamous cell carcinoma (SCC). BCC is associated with low mortality (and usually not included in general cancer statistics) due to has low metastatic potential. However, patients with SCC have a high risk of developing subsequent nodal metastases [3]. On the other hand, the most dangerous type of skin cancer is malignant melanoma, which lead to the death of patients in higher proportion due to the late detection of pathology and its higher risk to produce systemic metastases [2]. The process to diagnose skin cancer is accomplished by a dermatologist who perform a preliminary diagnosis by visually examining the PSL following the ABCDE (Asymmetry of the mole, Border irregularity, Color uniformity, Diameter and Evolving size, shape or color) rule [4]. After this examination, a biopsy is performed if the dermatologist suspects that the lesion is malignant. Then, a pathological analysis of the sample is carried out to assess the definitive diagnosis. There are several tools based on dermoscopic images and algorithms that implement the ABCD rule (without taking into account the evolving characteristics) to assist dermatologist in their clinical routine practice for PSL evaluation and classification [5,6]. Nevertheless, the current methodologies are not accurate enough, giving as a result several false positives and negatives. To avoid unnecessary surgical procedures, because of the uncertainty in the current diagnoses, new methods to improve skin cancer diagnosis must be investigated.

In recent years, a non-invasive, non-ionizing and label-free imaging modality is arising in the medical field: hyperspectral imaging (HSI). This imaging modality can combine digital imaging with spectroscopy methods, providing increased spectral properties of a captured scene within and beyond the visual range of the electromagnetic spectrum [7,8]. In a hyperspectral (HS) image, each pixel contains the so-called spectral signature of the material/substance located in its corresponding spatial coordinates. It has been demonstrated that quantitative information of tissue physiology can be extracted through the spectral signature analysis [9]. The fundamentals of this technology and the instruments developed for capturing such type of data for in-vivo applications in the medical field have been widely studied [10]. However, there are few research dealing with the use of HSI for in-vivo skin cancer detection as presented in the review performed by Johansen et al. [11].

In the study carried out by Tomatis et al. authors had the goal of diagnosing melanoma lesions using a classifier based on a multilayer perceptron neural network model [12]. They employed a multispectral (MS) acquisition system (SpectroShade^®^) able to capture MS images of 15 spectral bands between 483 and 950 nm. With such system they generated an in-vivo skin PSL database of 1391 MS images (including 184 melanomas) from 1278 patients. The reported sensitivity and specificity results in the test set (including 306 non-melanoma and 41 melanoma lesions) were of 80.5% and 77.1%, respectively. Other commercial MS systems have been developed to assist in the detection of melanoma, such as SIAscope/SIAscopy [13] or MelaFind [14,15,16]. First, SIAscope/SIAscopy was capable to capture 8 bands in the 400–1000 nm spectral range, obtaining a sensitivity of 82.7% and a specificity of 80.1% for melanoma identification in a dataset composed by 52 melanomas and 296 non-melanoma PSLs [13]. Second, MelaFind is able to acquire MS images composed by 10 bands in the spectral rage comprised between 430 and 950 nm, being used in several research studies. In [14], Elbaum et al. reported sensitivity and specificity values of 100% and 84%, respectively, under a leave-one-out cross-validation procedure using a database composed by 63 melanomas and 183 melanocytic nevus. In [15], Monheit et al. achieved a 98.4% of sensitivity and 9.9% of specificity in a prospective multicenter study where a dataset conformed by 127 melanomas and 1505 non-melanoma lesions was generated. In [16], Fink et al. performed an observational study where 360 PSLs (3 melanomas and 357 excised and non-excised non-melanoma lesions) were captured, achieving a sensitivity and specificity values of 100% and 68.5%, respectively. Finally, Song et al. performed a paired comparison between MelaFind and a reflectance confocal microscopy system to differentiate between melanoma and non-melanoma in a small sample size database composed by 4 melanomas and 51 non-melanoma lesions [17]. The results obtained showed a superiority of the confocal microscopy system (sensitivity of 85.7% and specificity of 71.4%) respect to the MelaFind system (sensitivity of 66.7% and specificity of 25%).

Other research works developed customized classification frameworks to automatically differentiate between melanoma and non-melanoma lesions by using an HSI system based on a spatial scanning HS camera (ImSpector V8E, Specim, Oulu, Finland) [18,19,20]. These studies employed HS images composed by 124 bands in the spectral range of 380–780 nm, obtaining, in the most recent clinical trial, sensitivity and specificity values of 96% and 87%, respectively, with a database composed by 24 melanomas and 110 non-melanoma lesions [20].

Regarding to the discrimination between malignant and beings PSLs, the study of Stamnes et al. employed a MS acquisition system that captured 10 bands in the 365–1000 nm spectral range [21]. They reported sensitivity and specificity results of 97% in both metrics using a test set conformed by 35 malignant and 120 benign PSLs.

Despite these state-of-the-art works and commercial systems available for assisting in the skin cancer diagnosis using mainly MS imaging for melanoma and non-melanoma discrimination, there are still room for improvements and investigations using HSI for malignant and benign PSL discrimination, providing higher number of spectral bands in larger spectral ranges.

In this sense, the main goal of this research is the development of a classification framework based on HS image segmentation and supervised classification by employing a customized dermatologic HSI system (developed by this research group) able to capture real-time HS data of in-vivo PSLs composed by 125 bands in the 450–950 nm spectral range. This preliminary study aims to demonstrate, as a proof-of-concept, the potential use of HSI technology to assist dermatologists in the discrimination of benign and malignant PSLs (including both NMSC and melanoma lesions) during clinical routine practice using a real-time and non-invasive hand-held device. To the best of our knowledge, this is the first work focused in using snapshot HS cameras within the visual and near-infrared (VNIR) range to segment and classify among benign and malignant PSLs using only spectral information.

## 2. Materials and Methods

### 2.1. Hyperspectral Dermatologic Acquisition System

**Figure 1:**
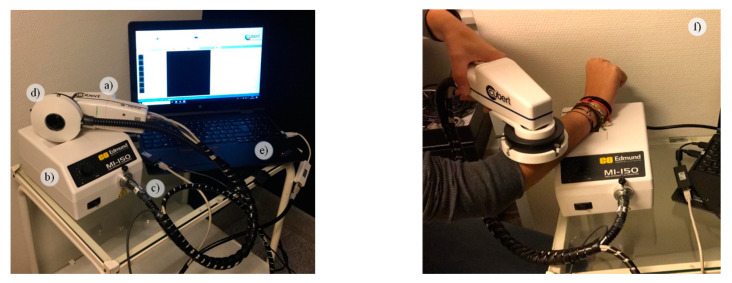
HS dermatologic acquisition system. (**a**) HS snapshot camera; (**b**) QTH (Quartz-Tungsten Halogen) source light; (**c**) Fiber optic ring light guide; (**d**) 3D printed customized dermoscopic contact structure attached to the ring light; (**e**) Acquisition software installed onto a laptop; (**f**) System employed during a data acquisition campaign.

The HS dermatologic acquisition system used in this work for the assistance in the diagnosis of PSLs is a custom development described in detail in [22]. The system is composed by a snapshot HS camera (Cubert UHD 185, Cubert GmbH, Ulm, Germany) capable of capturing HS data in the visual and near-infrared (VNIR) spectral range from 450 to 950 nm, having a spectral resolution of 8 nm (125 spectral bands) and a spatial resolution of 50 × 50 pixels (pixel size of 240 × 240 µm) (Figure 1a). This camera has coupled a Cinegon 1.9/10 (Schneider Optics Inc., Hauppauge, NY, USA) lens with a F-number of 1.9 and a focal length of 10.4 nm. The acquisition system employs a 150 W QTH-based (Quartz-Tungsten Halogen) illumination system (Dolan-Jenner, Boxborough, MA, USA) (Figure 1b) coupled to a fiber optic ring light guide to obtain cold light emission in the skin surface, avoiding the high temperatures produced by the halogen lamp (Figure 1c). The illumination system is attached to the HS camera through a 3D printed customized dermoscopic contact structure where the skin contact part is a dermoscopic lens with the same refraction index as the human skin (Figure 1d). The HS dermatologic system can capture HS images, with an effective area of 12 × 12 mm, with an acquisition time of ~250 ms. This system is connected to a laptop where the acquisition software is executed (Figure 1e). Figure 1f shows and example of the use of the developed HS dermatologic acquisition system during a clinical data acquisition campaign at the University Hospital Doctor Negrin of Las Palmas de Gran Canaria (Spain).

### 2.2. Study Design and HS Dataset Description 

The HS dermatologic acquisition system was employed to obtain an HS in-vivo human PSL database to evaluate the efficiency of HS images to discriminate between benign and malignant lesions. The data acquisition campaign was performed from March 2018 to June 2019. Several types of PSLs from different parts of the body were captured from 116 subjects in two different hospitals, the Hospital Universitario de Gran Canaria Doctor Negrín and Complejo Hospitalario Universitario Insular - Materno Infantil (Spain). The study protocol and consent procedures were approved by the *Comité Ético de Investigación Clínica-Comité de Ética en la Investigación (CEIC/CEI)* from both hospitals. Written informed consent was obtained from all subjects.

**Figure 2:**
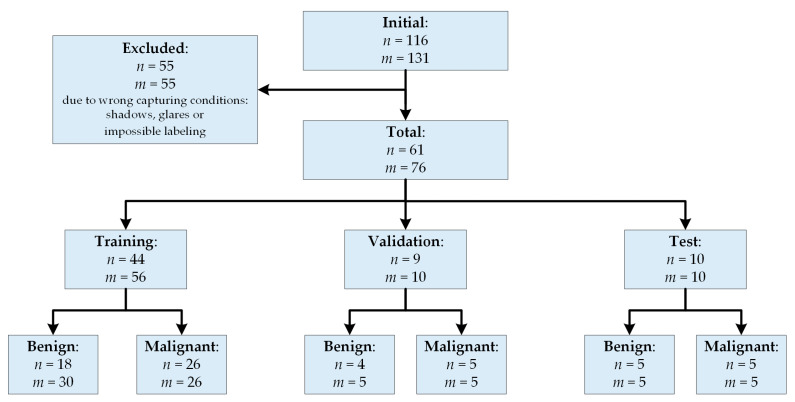
Patient/image flow scheme in this study. ***n***: number of patients; ***m***: number of HS images.

After a preliminary analysis of the captured data, 55 subjects/images were removed from the database due to the PSLs were located in areas extremely difficult to be captured (e.g., shoulders, nose, chin, and other parts of the face) and, hence, the HS images were not recorded in optimal conditions. The dermoscopic lens had no complete contact with the skin surface, producing shadows or glares in the images and, consequently, it was impossible to perform reliable image calibration or PSL labeling on captured HS images. The final database was composed by 76 images from 61 subjects as shown in Figure 2, where it is also included the training, validation and test set distribution of this preliminary study.

In addition to the HS image, a standard digital dermoscopic camera (3Gen Dermlite Dermatoscope, 3Gen Inc., San Juan Capistrano, CA, USA) was employed to capture conventional RGB images of 3000 × 4000 pixels (pixel size of 6.6 × 6.6 µm) of the same PSL for dermatologist evaluation. Suspected malignant lesions were diagnosed through histological assessment.

#### 2.2.1. HS Labeled Dataset

**Figure 3:**
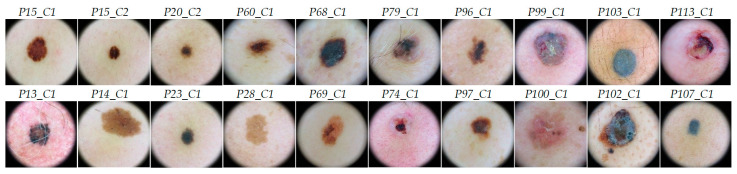
RGB images obtained with the digital dermoscopic camera with their correspondent image ID above. The first row shows the validation set images and the second row the test set images.

A labeled dataset was created employing the HS images by assigning to certain pixels the diagnostic class of the PSL obtained from the dermatologist/pathologist assessment. This assignation was performed by using a semi-automatic labeling tool based on the SAM (Spectral Angle Mapper) algorithm. This algorithm determines the spectral similarity between two spectral signatures, where lower spectral angle values indicate higher similarity among both spectral signatures [23]. The semi-automatic labeling tool allows labeling the most similar pixels in the image with respect to a reference pixel, which was manually selected and identified to belong to a certain class. Only pixels with high confidence to belong to a class were labeled. This tool has been already employed to label HS images in-vivo brain surface for brain tumor classification [24]. After performing the labeling of the entire database, a total of 15,961 pixels were used for the classification experiments employing machine learning algorithms. The data were labeled in two different classes: *Benign* and *Malignant*. Concretely, the labeled dataset was composed by 61 patients, but two of them have different lesions captured where one lesion belongs to the benign class and the other lesion belong to the malignant class. Table 1 shows the number of patients, images and labeled pixels per class. Figure 3 shows some RGB dermoscopic images obtained by using the digital dermoscopic camera. The HS images corresponding to these image IDs were employed as validation and test sets in the experimental setup.

#### 2.2.2. HS labeled Data Partition

The HS labeled dataset of PSL spectral signatures was employed to train, validate and test the developed classification algorithms. The validation process was performed using a patient stratified assignment where the labeled data were divided into three independent sets: *test*, *validation* and *training*. The test set was composed by labeled data from 10 images from 10 patients with 2472 pixels. The validation set was formed by labeled data from 10 images from 9 patients, having 1931 pixels and, the training set was composed by the remaining labeled data of 56 images from 44 patients, formed by 11,558 pixels. Table S1 of the supplementary material shows the details of the dataset. Hence, in this dataset different patients were used for training, validation and test.

### 2.3. HS Dermatologic Data Pre-Processing

The HS data were pre-processed to homogenize the spectral signatures among the different patients and data campaigns. Three main steps form the pre-processing chain: radiometric calibration, noise filtering and normalization.

First, a radiometric calibration was performed to the raw HS image (*RI*) employing a white reference image (*WI*), captured from a white reference tile able to reflect the 99% of the incident light, and a dark reference image (*DI*), recorded by having the light turned off and the camera shutter closed. *WI* and *DI* were acquired before the PSL data acquisition and in the same illumination conditions. The calibrated image (*CI*) was obtained following Equation (1).

(1) $$CI=\frac{RI-DI}{WI-DI}$$

Second, in order to reduce the spectral noise found in the spectral signatures, the first 4 bands and the last 5 bands were removed due to the HS sensor low response in such bands. Moreover, the HS data was filtered using a smooth filter for reducing the spectral noise in the remaining spectral bands. The final spectral signature was formed by 116 bands. In the final step, a normalization was applied to each spectral signature to range the data between 0 and 1 with the goal of homogenizing its amplitude, thus avoiding the subsequent processing methods to be affected by the amplitude differences caused by non-uniform illumination conditions. In this sense, only the shape of the spectral signature will be considered.

**Figure 4:**
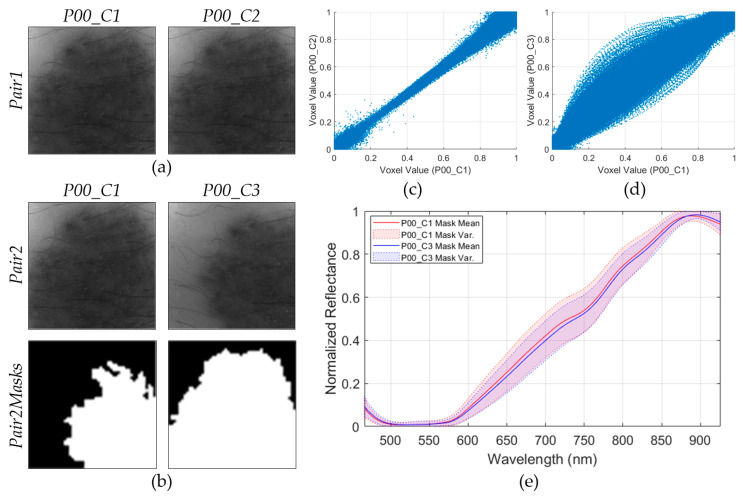
Repeatability results of the HS dermatologic acquisition system. (**a**) Gray-scale representation of the two consecutive HS images from the same PSL (*Pair1*). (**b**) Gray-scale representation of the two HS images from the same PSL but captured in different spatial positions (*Pair2*) and binary mask for the segmentation of the PSL pixels (*Pair2Masks*), where white pixels represent the PSL pixels. (**c**) Scatterplot of voxel values of *Pair1*. Repeatability results: $RD_{mean}=9.52\%$. (**d**) Scatterplot of voxel values of *Pair2*. Repeatability result: $RD_{mean}=23.68\%$. (**e**) Mean and variance of the segmented PSL pixels from *Pair2*.

In order to assess the repeatability of the HS dermatologic system, two consecutive HS images of the same lesion in the same exact location (P00_C1 and P00_C2 in Figure 4a), called Pair1, were employed. Moreover, another pair of images (Pair2) of the same lesion but captured at different spatial positions (P00_C1 and P00_C3 in Figure 4b) was employed. In order to segment the PSL pixels of the Pair2 images, a binary mask was created for each image, as can be seen in the last row of Figure 4b, where the white pixels in the Pair2Masks represent the selected PSL pixels.

The main goal of this analysis is the evaluation of the possible systematic errors that can be found in the acquisition system, and also to verify the spectra repeatability when images of the same scene are obtained with subtle different conditions. To perform the repeatability analysis, three experiments were proposed: repeatability of Pair1, repeatability of Pair2, and spectral mean and variance analysis of the Pair2 PSL pixels using the Pair2Masks.

The first experiment evaluates the repeatability of Pair1, where two consecutive HS images of the same lesion (P00_C1 and P00_C2) were captured in the same exact position. To analyze the differences between these images, a scatterplot was employed Figure 4c), where the voxel values of each HS image of Pair1 are represented (290,000 voxel pairs). The voxel value represents the reflectance of the light in a certain pixel of the HS image at a certain wavelength. Ideally, the scatterplot should be a straight line, which indicates that each voxel pairs encloses the same exact information. As it can be seen, when two consecutive images are compared, the scatterplot is similar to the ideal situation.

In the second experiment, the scatterplot of the HS images of Pair2 (same lesion but different spatial positions) was generated (Figure 4d). In this case, the scatterplot does not show a straight line, but several voxel pairs have the same information because it is the same injury. For this reason, a third experiment based on a visual comparison of the spectral signatures of the PSL pixels of Pair2 was performed. The PSL pixels were segmented using the Pair2Masks, and the mean and variances of the preprocessed spectral signatures of such pixels were represented in Figure 4e. As it can be seen, the mean and variances of both images are quite similar, suggesting that the HS dermatologic system is reliable even when capturing data from the same lesion but in different conditions.

Additionally, the absolute relative difference percentage (RD) was obtained for the first and second experiment using Pair1 and Pair2, respectively. This metric is employed to measure the repeatability of a system and it is computed using Equation (2), where x and y represent the data from the HS image pair. Lower values of RD represent higher similarity. In the first experiment, Pair1 obtained a ${\mathrm{RD}}_{\mathrm{mean}}$ of 9.52%, while in the second experiment (Pair2) the result was worsened due to the differences in the spatial coordinates of the PSL (${\mathrm{RD}}_{\mathrm{mean}}=23.68\%$).

(2) $$RD\left(\%\right)=\frac{abs\left(x-y\right)\cdot 100}{\left[mean\left(x\right)+mean\left(y\right)\right]/2}$$

### 2.4. HS Dermatologic Segmentation Framework

**Figure 5:**
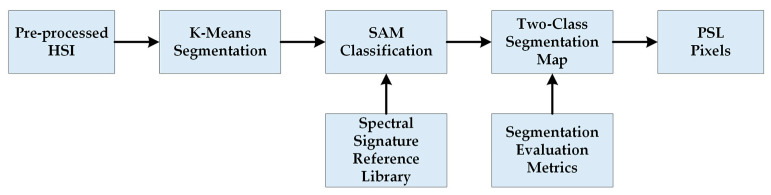
Block diagram of the HS dermatologic segmentation framework.

In this section, a processing framework to automatically segment the captured HS image into normal skin and PSL pixels based on an unsupervised segmentation algorithm is proposed (Figure 5). The PSL pixels identified in this framework will be afterwards classified into benign or malignant classes by the classification framework. The K-means clustering algorithm was selected to perform the segmentation as it is a well-established algorithm that provides a good delimitation of the different areas presented in an HS image scene [25]. This algorithm divides an input HS image into K different clusters for a previously selected K value. However, the identification of each cluster is not associated to any pre-established class, so the segmentation maps only represent relevant spectral differences. In this framework, first, the evaluation of the optimal K value for this application is performed. Different clustering evaluation methods were employed to determine the optimal K value, such as Silhouette [26], Calinski Harabasz [27] and Davies Bouldin [28] methods. The training dataset was used to find the optimal *K* value. Table 2 shows the minimum and maximum K values obtained from the different methods, where the most frequent value to segment the image is two. Considering this result, the range between two and seven clusters will be evaluated to compare the results and select the K value that provides the best result.

**Figure 6:**
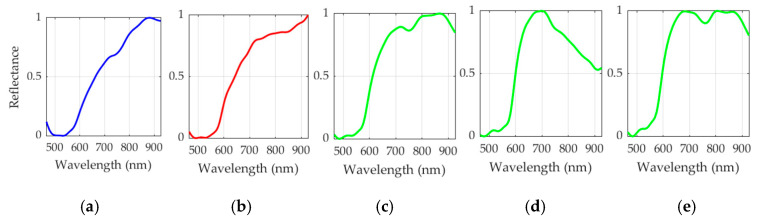
Reference spectral signatures included in the skin/PSL library. Benign (**a**) and malignant (**b**) PSL spectral signatures. (**c**–**e**) Three different normal skin spectral signatures of the training dataset.

**Figure 7:**
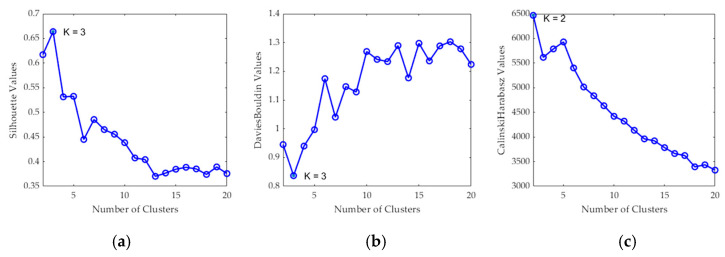
Clustering evaluation to segment the normal skin training dataset. Results of the optimal cluster number evaluation using the following methods: (**a**) Silhouette (maximum K indicates optimal value), (**b**) Davies Bouldin (minimum K indicates optimal value) and (**c**) Calinski Harabasz (maximum K indicates optimal value).

After the *K* value evaluation, a two-class segmentation map is generated where the PSL and the normal skin pixels are identified considering the information of each cluster of the segmentation map, using the SAM algorithm. In order to perform the SAM comparison, a spectral signature reference library of normal skin and PSL data was created, employing only the spectral signatures of the labeled training set in order to avoid the inclusion of validation or test HS images in the reference library (see Section 2.2.2). This library contains five different spectral signatures: three from normal skin, and two from malignant and benign PSLs (see Figure 6). These reference spectral signatures were obtained computing the average of the labeled data per class. The normal skin data were divided into three groups using the K-means clustering algorithm, where the number of clusters employed was selected after evaluating the results using the Silhouette, Calinski Harabasz and Davies Bouldin methods. The Silhouette and Davies Bouldin methods indicate that the optimal number of clusters to segment the normal skin data was three; taking into account the smallest index value achieved in Figure 7a,b. Instead, for Calinski Harabasz method the optimal *K* value was two, considering the highest index value reached in Figure 7c. Taking into account these results, the selected number of clusters to segment the training set was established in three. These reference spectral signatures were employed to automatically identify the PSL pixels through the SAM algorithm, which will be next considered as input for the supervised classification.

**Figure 8:**
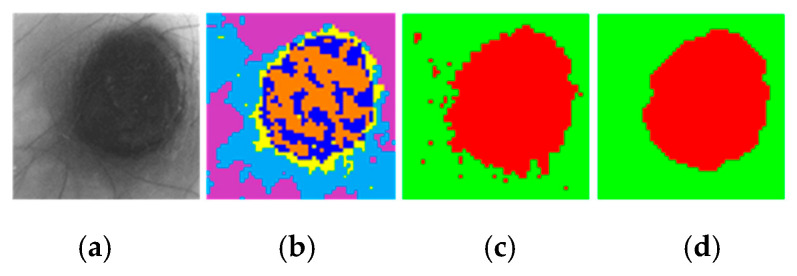
HS dermatologic segmentation example. (**a**) Gray-scale image. (**b)** Segmentation map using five clusters (colors are randomly assigned). (**c**) Two-class segmentation map obtained after comparing the five centroids with the reference library using the SAM algorithm (red indicates PSL and green normal skin). (**d**) Two-class segmentation map after applying morphological closing operation.

For the computation of the SAM algorithm, two different methods were employed to generate the two-class segmentation maps. The first method (called *per centroid*) compared the centroid from each cluster of the segmentation map with the spectral signatures of the reference library. In this method, the most similar spectral signature to each centroid was assigned to a certain class (PSL or normal skin). The second method (called *per pixel*) compared each pixel in a certain cluster with the spectral signatures of the reference library and computed the sum of the resulting SAM values. Then, the smallest sum in each centroid is assigned to a certain class (PSL or normal skin). Finally, a morphological closing operation based on dilatation followed by erosion was applied to the two-class segmentation map in order to remove small isolated regions and to obtain a better representation of the lesion. Figure 8 shows an example of a segmentation, where Figure 8a shows the gray-scale image and Figure 8b shows the segmentation map of an HS image using five clusters, where the colors have no physical meaning. Figure 8c shows the classification map obtained after applying the SAM methodology, while Figure 8d shows the same two-class segmentation map after the morphological post-processing. In these maps, normal skin and PSL pixels are represented in green and red colors, respectively.

Finally, these results were compared with the ground truth maps of the validation dataset using segmentation evaluation metrics to select the most appropriate K value and SAM comparison method. The PSL pixels were used as input for the supervised classification in the complete processing framework.

### 2.5. HS Dermatologic Classification Framework

**Figure 9:**
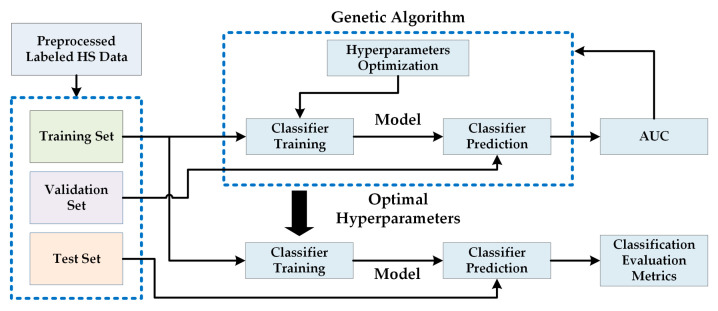
Proposed block diagram of the HS dermatologic classification processing framework.

The HS dermatologic classification framework developed in this work is based on a supervised classification with an automatic fine tuning of the classifier hyperparameters employing an optimization algorithm. The pre-processed HS labeled dataset was employed to find the most suitable classification model using the data partitions presented in Section 2.7.2. Figure 9 shows the block diagram of this processing framework, where a Genetic Algorithm (GA) was employed to optimize the hyperparameters of the supervised classifier using the training and validation sets. The area under the curve (AUC) was used for the evaluation of the validation results (see Section 2.7). After finding the optimal hyperparameters, the classifier is trained with the training set and evaluated with the test set, obtaining the final evaluation metrics (see Section 2.7.2). The supervised classification algorithms evaluated in this work are Support Vector Machines (SVMs), Random Forest (RF) and Artificial Neural Networks (ANNs) [29]. These classifiers have been commonly used for the classification of HS data in the literature, especially in medical HSI applications [10].

#### 2.5.1. Support Vector Machine (SVM) Classifier

The SVM classifier is a supervised classification algorithm [30]. Its objective is to find out the best hyperplane that allows separating the different data with a maximum margin. The SVM was selected because it has been proven in the literature to perform well with highly imbalanced training datasets [31], being also widely used for HS data classification in medical applications [32,33].

In this study, the linear, Radial Basis Function (RBF) and the sigmoid kernels were compared in performance for the SVM classifier. The optimal configuration of the SVM was adjusting by finding the optimal hyperparameters for each type of kernel. Table S2 in the supplementary material shows the detailed kernel functions and their hyperparameters. LIBSVM was used for the SVM classifier implementation [34].

#### 2.5.2. Random Forest (RF) Classifier

The RF algorithm is an ensemble learning method capable of constructing a set of decision trees able to classify new data samples in a specific class by voting the decision trees predictions [35]. RF can be optimized by establishing the most suitable number of trees in the classification model. This classifier was selected for evaluation since it has shown good performance in classifying medical HS data [36]. To implement RF classifier, the MATLAB^®^ (The MathWorks Inc., Natick, MA, USA) Machine Learning ToolBox^TM^ was employed.

#### 2.5.3. Artificial Neural Network (ANN) Classifier

The ANN classifier imitates the human brain process to transfer information [37]. The optimization of the ANN model is performed with the objective of identifying the best number of *neurons* for each layer. This classifier has been also employed in the literature to process HS medical data [38]. The ANN architecture employed in this work was composed by four layers. Thus, four parameters were optimized in this classifier. To implement the ANN classifier, the MATLAB^®^ Deep Learning ToolBox^TM^ was used.

#### 2.5.4. Genetic Algorithm (GA)

The GA is a non-linear global optimization algorithm proposed by Holland et al. in the late 1960s [39]. The theory of the biological evolution (proposed by Charles Darwin) is the basis of this algorithm (survival of the fittest, crossover, mutation, etc.) [40]. GA has been used in several types of optimization problems due to its straightforwardness and robustness [41,42,43]. The GA implementation used in the experiments performed in this work was based on the MATLAB^®^ Global Optimization ToolBox^TM^.

### 2.6. HS Dermatologic Framework for In-Situ Clinical Support

**Figure 10:**
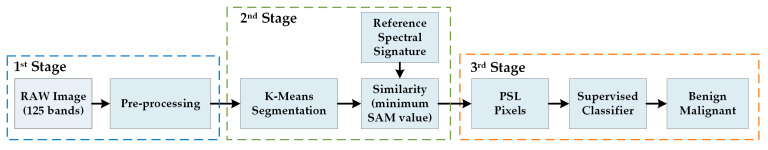
Block diagram of the HS dermatologic framework for in-situ clinical support.

The complete processing framework is composed by three stages based on the three previously presented processing frameworks with the aim of supporting in-situ diagnosis of PSLs during clinical routine practice. Figure 10 shows the block diagram of the complete processing framework where the different stages are interconnected. The first stage performs the pre-processing chain of the incoming raw HS image captured by the acquisition system. This pre-processed HS image is the input of the second stage, where the segmentation between PSL and normal skin pixels is performed. In the last stage, the pixels identified as PSL are classified, providing the dermatologist with the PSL class (Benign or Malignant) and the probability value of belonging to such class.

### 2.7. Evaluation Metrics

#### 2.7.1. Segmentation Evaluation Metrics

Overlap-based metrics were employed to evaluate the segmentation quality achieved by the K-means algorithm, comparing the segmented image (*SI*) against the ground truth (*GT*). Dice similarity coefficient measures the match between two images and is equal to twice the intersection divided by the sum of the both images Equation (3) [44]. Jaccard similarity coefficient measures the similarity between the *GT* and *SI*, being defined as the intersection over the union of the two images Equation (4) [45]. These metrics are the most used in image segmentation evaluation and can be expressed using the definition of true positives (TP), false positives (FP), and false negatives (FN). Dice and Jaccard coefficients are similar metrics and both measurements have a value range in [0, 1]. However, Jaccard coefficient penalizes misclassifications more than Dice coefficient. For this reason, only the Jaccard coefficient will be employed in this work to select the optimal number of clusters (K) and the best segmentation methodology.

(3) $$Dice=\frac{2\left|SI\cap GT\right|}{\left|SI\right|+\left|GT\right|}=\frac{2TP}{2TP+FP+FN}$$

(4) $$Jaccard=\frac{\left|SI\cap GT\right|}{\left|SI\cup GT\right|}=\frac{TP}{TP+FP+FN}$$

#### 2.7.2. Classification Evaluation Metrics

The receiver operating characteristic (ROC) curve was employed to find the optimal hyperparameters of the supervised classifiers, finding the best performance using the AUC (Area Under the Curve) metric. The ROC curve represents how sensitivity changes with varying specificity and is used on binary classifications to determine if one variable is more predictive than another [46]. Equations (5) and (6) presents the sensitivity and specificity expression where TP is the number of true positives, FN the number of false negatives, TN the number of true negatives, and FP the number of false positive.

In order to evaluate the results obtained for the optimized classifier (in both validation and test sets), the accuracy (ACC) metric was computed (see Equation (7)). For this particular application, when the evaluation of the complete framework is performed, the ACC is equivalent to the sensitivity of the classifier due to the pixels to be classified only belong to one class and there are no *TNs* and *FPs*.

(5) $$Sensitivity=\frac{TP}{TP+FN}$$

(6) $$Specificity=\frac{TN}{TN+FP}$$

(7) $$ACC=\frac{TP+TN}{TP+TN+FP+FN}$$

## 3. Experimental Results and Discussion

This section will present the validation and test results achieved in the independent experiments of the segmentation and classification frameworks, as well as the results obtained when employing the complete processing framework for in-situ clinical support.

### 3.1. HS Dermatologic Segmentation Framework Results

The proposed segmentation framework has the goal to select only PSL pixels in an HS image to reduce the data that will be sent to the classification stage and, consequently, decrease the computational cost of SVM classifier, performing a two-class classification. In this framework, the validation dataset presented in Section 2.2.2 was employed. Thus, 10 HS validation images from 8 different patients were evaluated with two methods (*per centroid* and *per pixel*) based on the K-means and the SAM algorithms, using different K values to find out which combination of method and number of clusters offers the best results.

**Figure 11:**
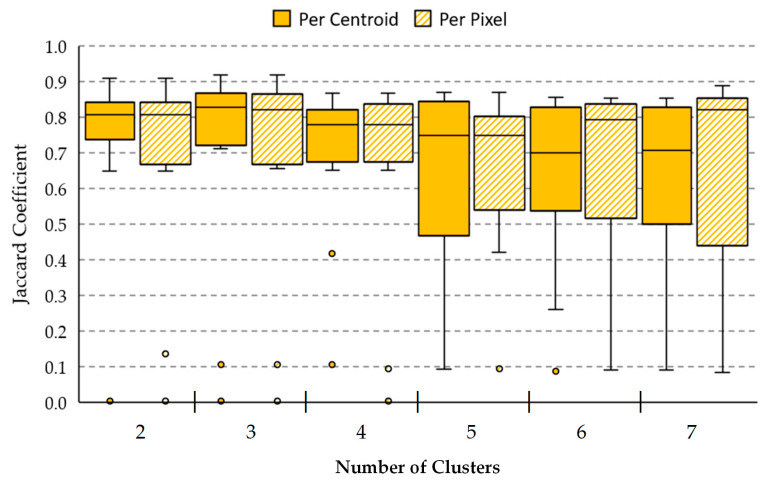
Comparison between *per centroid* and *per pixel* methods using different number of clusters for the validation data using the Jaccard coefficient. The box boundaries represent the IQR of the results. Central bars and error bars depict median and minimum/maximum values of Jaccard coefficient, respectively. The small dots outside the minimum/maximum values represent the outliers of the Jaccard coefficient found in each method.

**Figure 12:**
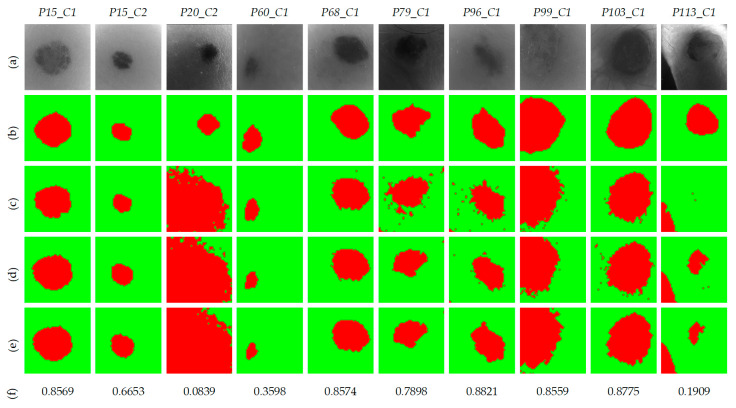
Two-class segmentation maps of the validation database using the *per pixel* method. (**a**) Gray-scale images. (**b**) Ground-truth maps. (**c**) Results with K=3. (**d**) Results with K=7. (**e**) Results with K=7 and morphological post-processing. (**f**) Jaccard coefficient values of the results with K=7 and morphological post-processing.

Figure 11 shows the boxplot results of the Jaccard coefficient metric using the 10 HS validation images for each method (per centroid and per pixel) using different number of clusters in the range 2 ≤ K ≤ 7, as was established in Section 2.4. Table S3 in Supplementary Material details the Jaccard coefficient obtained for each one of the HS validation images, from where the boxplot was generated. In the figure, the boxes boundaries represent the interquartile ranges (IQR), which regards the results of the validation set comprised between the first quartile (Q1, 25th percentile) and the third quartile (Q3, 75th percentile). The central bars represent the median result values (Q2, 50th percentile), while the error bars depict minimum and maximum values of the Jaccard coefficient for such method excluding any outliers. The outlier values are represented in the plot with the small dots. Attending to the boxplots, K=2 with the *per centroid* method offers the best IQR value with a median of 0.81, while K=3 and K=7 provide the best median results in both methods higher than 0.82, also representing a reduced (IQR) for K=3. However, it should be noted that most of the results in the boxplot present one or two outliers (represented with small dots), where their Y positions show the Jaccard value for a specific HS image of the validation set in such method (see detailed values in Table S3 of the supplementary material). This abnormal distance from the other values is produced due to images *P20_C2* and *P113_C1* were not captured in optimal conditions, producing shadows or glares in the HS images (see gray-scale images in Figure 12). Considering these outliers and analyzing the two-class segmentation maps when K=2 and K=3 are used, no PSL pixels are detected in P113_C1 image. On the contrary, it is observed the results using K=7 offer a better segmentation of the PSLs. For example, P113_C1 is better segmented than the K=2 and K=3 results (as will be shown in Figure 12), allowing PSL classification to be performed by the supervised classifier. In addition, using K=7, the per pixel method provides a better median value (0.82) than the per centroid method (0.71), representing an improvement of 11%. For this reason, we selected K=7 with the per pixel method as the most suitable configuration for the overall framework.

Figure 12 shows the qualitative results obtained in the segmentation framework using the *per pixel* method. Figure 12a shows the gray-scale images for each HS validation cube, while Figure 12b shows the ground-truth, where the PSL has been manually segmented by an expert. Figure 12c,d show the two-class segmentation maps obtained with K=3 and K=7, respectively. It is observed that the results in both cases are very similar. Nonetheless, in the case of *P113_C1*, using K=7 the qualitative results are better than the other case. Finally, Figure 12e shows the two-class segmentation maps after performing a morphological closing operation to remove small isolated regions of PSL pixels, ensuring that in the next classification stage, only PSL pixels will be employed. The PSL area is clearly identified in almost all images, except in images *P20_C2, P60_C1*, and *P113_C1*, achieving an average Jaccard value of 0.82.

**Figure 13:**
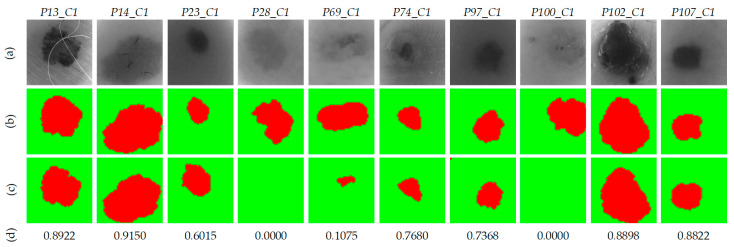
Two-class segmentation maps of the test database using *per pixel* method with K=7. (**a**) Gray-scale images. (**b**) Ground-truth maps. (**c**) Results with morphological post-processing. (**d**) Jaccard coefficient values of the results with morphological post-processing.

Taking into account the results obtained, it has been concluded that the *per pixel* method with K=7 and morphological post-processing provides the best results with the validation database. Next, the evaluation of the test database, composed by 10 HS images from 10 different patients, using the selected method was performed to validate the algorithm for the automatic identification of the PSL pixels. Figure 13 shows the qualitative and quantitative results for each HS test image. The resulting two-class segmentation maps after applying the morphological post-processing are shown in Figure 13c, and below, their respective Jaccard coefficients. It is worth noticing that the results obtained in images *P13_C1*, *P14_C1*, *P23_C1, P74_C1, P97_C1*, *P102_C1,* and *P107_C1*, the PSL areas are clearly identified, achieving an average Jaccard value of 0.81.

**Figure 14:**
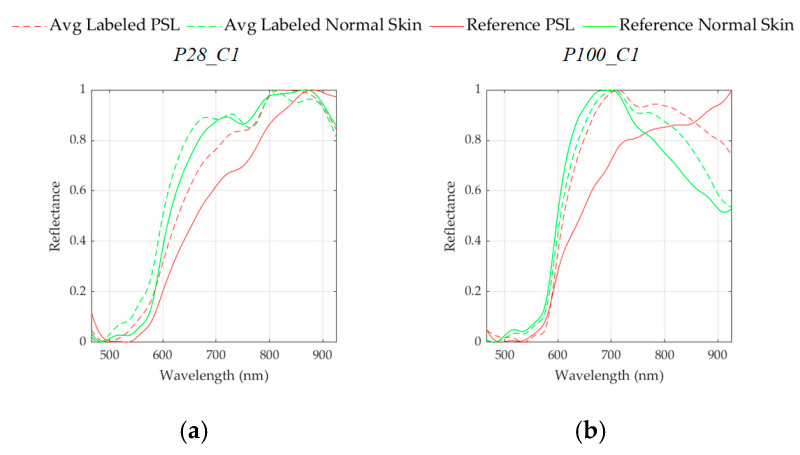
Average spectral signatures of the labeled PSL (dashed red line) and normal skin (dashed green line) pixels, and reference spectral signatures of PSLs (red line) and normal skin (green line). (**a**) *P28_C1* (benign PSL). (**b**) *P100_C1* (malignant PSL).

Nonetheless, in *P69_C1* image, a small area of the PSL pixels was identified with a Jaccard value of 0.10. However, this area corresponds with the center of the lesion, enabling the more relevant pixels of the PSL to be processed by the next classification stage. On the other hand, in images *P28_C1*, and *P100_C1* the segmentation process did not detect any PSL pixel. After analyzing the spectral signatures of these images and comparing them with the spectral signatures of the reference library, it was observed that the PSL spectral signatures of both images were very similar to the normal skin references. This phenomenon can be observed in Figure 14, where a comparison between the reference spectral signatures and the average of the PSL and normal skin pixels was performed. In the case of *P28_C1* (Figure 14a), the PSL was diagnosed as a benign lesion; however, the average spectral signature of the PSL is more similar to the normal skin references than to the benign reference. In the case of *P100_C1* (Figure 14b), the PSL was diagnosed as a malignant lesion, but the average spectral signature of the PSL is more similar to the normal skin references than to the malignant reference. These results suggest the necessity of increasing the HS database to improve the spectral signature reference library with the wide variability of PSLs and normal skin types. In addition to these results, Figure S1 in the Supplementary Material shows the average spectral signature comparison for the rest of the HS test images.

### 3.2. HS Dermatologic Classification Framework Results

In this section, the experimental results obtained in the classification of the labeled samples of the PSLs from the HS database employing the different classifiers are presented. Table 3 shows the AUC results obtained with each supervised classifier using the default and the optimal hyperparameters to classify the validation dataset presented in Section 2.2.2. The default hyperparameters were established by [34,47,48] and the optimal values were obtained by the experiments performed using the GA.

**Figure 15:**
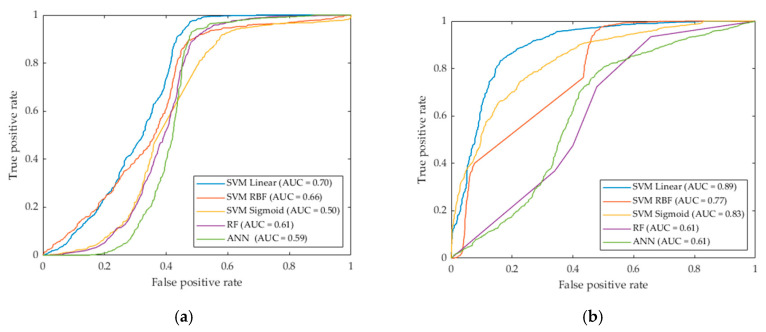
ROC curves for validation classification results obtained with the five classifiers. (**a**) Classification results with default parameters. (**b**) Classification results with optimized hyperparameters.

As it can be seen in the results, the optimized SVM Linear algorithm achieved the best AUC (0.89), followed by the SVM Sigmoid and SVM RBF algorithms (0.83 and 0.77, respectively). In addition to these results, Figure 15 shows the ROC curves obtained with each classifier with and without hyperparameters optimization. In this figure it is possible to observe the differences between the curves, where SVM Linear, Sigmoid and RBF classifiers improve the results after the optimization. Nevertheless, RF and ANN classifiers show no relevant improvement in the results. Taking into account these results, the SVM Linear was selected for the classification of the PSLs to complete the processing framework shown in Section 2.6, achieving a sensitivity of 96.7%.

**Figure 16:**
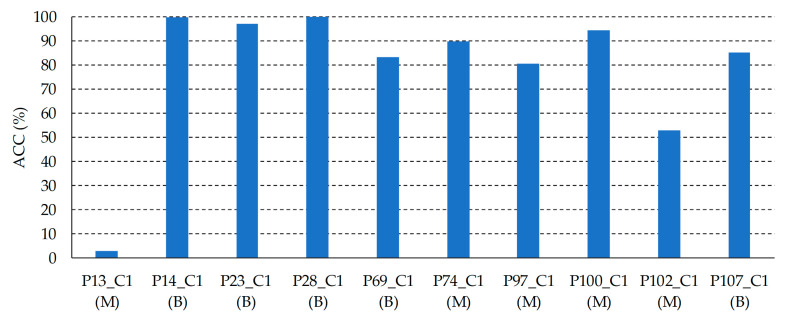
Test classification ACC results obtained with the SVM Linear classifier. Below each patient ID, the correct diagnosis of the PSL is presented. B: Benign; M: Malignant.

**Figure 17:**
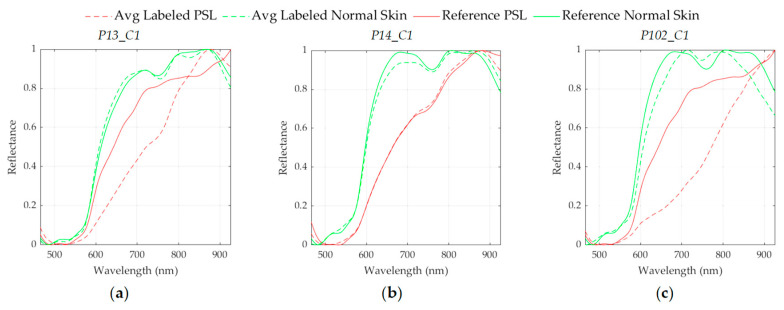
Average spectral signatures of the labeled PSL (dashed red line) and normal skin (dashed green line) pixels, and reference spectral signatures of PSLs (red line) and normal skin (green line). (**a**) *P13_C1* (malignant PSL). (**b**) *P14_C1* (benign PSL). (**c**) *P102_C1* (malignant PSL).

In order to assess the results obtained with the SVM Linear classifier optimized with the validation set, the classifier was evaluated on the test set. Figure 16 shows the ACC results of each HS test image, where it is possible to observe that 8 images were classified with an ACC higher than 80%, one image (*P102_C1*) was identified with a 53% of ACC, and only one HS image (*P13_C1*) was not correctly classified. As it can be seen in Figure 17, the average spectral signatures of the malignant lesions *P13_C1* and *P102_C1* are quite different from the reference spectral signatures of such classes (Figure 17a,c). On the contrary, *P14_C1* offers an excellent classification accuracy value, being its average spectral signature highly similar to the reference benign spectrum (Figure 17b). In this sense, it is possible that the skin cancer database requires more data and patients variability to generalize a classification model able to achieve higher accuracy. Summarizing, in the test set the classifier provided an average ACC of 78%, identifying correctly 9 PSLs and 1 PSL not correctly identified.

### 3.3. HS Dermatologic Overall Results

This section presents the results obtained with the fully HS dermatologic processing framework presented in Section 2.6. This framework is composed by the selected segmentation and classification algorithms which provided the best results in the previous analysis.

**Figure 18:**
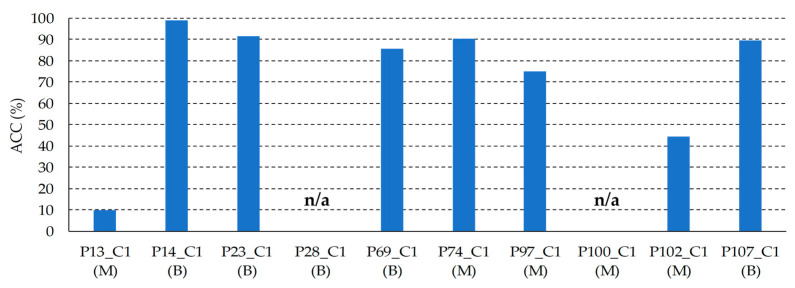
Test classification ACC results obtained with the SVM Linear classifier and with the pixel segmentation dataset. **n/a:** HS images without PSL pixels identified in the segmentation stage.

Figure 18 shows the ACC results for each HS test image after applying the segmentation and classification of the PSL pixels. On the one hand, in the images *P28_C1*, and *P100_C1*, no pixels were identified as PSL by the segmentation stage (see Section 3.1, Figure 13). Thus, the classification stage could not provide the identification of the pixels. In this case, the system will require asking the user a new acquisition of the PSL due to the non-optimal conditions of the captured HS image. On the other hand, the PSL image *P13_C1* achieved a very low accuracy in the identification of the lesion (10%), while image *P102_C1* obtained an accuracy of 45%. As explained in the previous section, the spectral signatures of these lesions are quite different from the reference spectra, indicating the need of an increased database where the inter-patient and inter-lesion variability were taken into account. The remaining HS test images (*P14_C1, P23_C1, P69_C1, P74_C1*, *P97_C1*, and *P107_C1*) provided competitive results in the identification of the PSL type with an average ACC of 85%.

Summarizing, using the proposed processing framework in this preliminary study, two of the HS test images were not evaluated due to non-optimal conditions of the acquisition procedure. In addition, another HS test image was not correctly identified due to the necessity of increasing the HS PSL database in order to better generalize the segmentation and classification models for the large diversity of PSLs and skin types. However, using a risk threshold of 40% for the discrimination of the malignant lesions, 7 of 8 evaluable HS test images (87.5%) were accurately classified according to the PSL pathological diagnosis. In this sense, the malignant PSLs with a higher accuracy than 40% will be considered that have a clear evidence of malignant behavior.

These preliminary results are highly promising due to the strict validation methodology employed is based on dividing the database into training, validation and test sets. In this sense, the test set is composed by data from patients not involved in the generation of the processing models. This guarantees the reliability of the achieved results without producing overfitting, which can provide optimistic accuracy results. In addition, the average execution time for the proposed HS dermatologic framework is ~500 ms, requiring ~220 ms to perform the pre-processing stage, ~135 ms for the segmentation stage and ~145 ms to execute the supervised classification. The implementation was performed using MATLAB^®^ in an Intel i7-4790K with a working frequency of 4,00 GHz and a RAM memory of 8 GB. Therefore, this preliminary study reveals the potential use of HSI as a non-invasive imaging modality for in-situ clinical support during the routine clinical practice.

In order to compare the results obtained in this preliminary study with the state-of-the-art, a summary table is shown in Table 4. It is worth noticing that our work cannot be directly compared to the most of the already published studies since our focus is to discriminate between benign and malignant PSLs, while the other research works are based on discriminating between melanoma and non-melanoma lesions. Besides, since the dataset used in each research is different, the comparative between different approaches is not fair. Nevertheless, we would like to present the most relevant state-of-the-art results.

In [12], the research of Tomatis et al. used a dataset of 1278 patients with 1391 images, where 184 lesions were melanomas. The dataset was divided into three sets, where the test set was composed by 347 images, including 41 melanomas. The sensitivity obtained was 80.4% with a specificity of 75.6%. Moncrieff et al. performed a discrimination between melanoma and non-melanoma lesions by using the multispectral SIAscope/SIAscopy system to generate a database composed by 52 melanomas and 296 non-melanomas, achieving a sensitivity and specificity of 82.7% and 80.1%, respectively [13]. The studies performed by Fink et al. [16] and Song et al. [17] were based on MelaFind system, achieving a 100% and 71.4% of sensitivity, respectively, but having a very low number of melanomas in the database (3 and 4 melanomas, respectively). However, the multicenter study of Monheit et al. [15] evaluated the MelaFind tool with a dataset of 1612 images (including 114 melanomas) and achieved a sensitivity of 98.2% but with a very low specificity (9.5%). In another study performed by Nagaoka et al. authors generated a database composed by 24 melanomas and 110 non-melanoma lesions using a HS system capable of obtaining 124 bands, achieving a sensitivity and specificity of 96% and 87%, respectively [20].

To the best of our knowledge, the only work found in the literature which deals with the discrimination between malignant and benign PSL was performed by Stamnes et al. [21]. In this work, two datasets were evaluated: a small dataset with 157 images (35 malignant and 39 benign); and a large dataset, which included lesions employed to train the system, composed by 712 images (80 malignant and 217 benign). The results were promising achieving sensitivity and specificity of 97% and 99%; and 97% and 93% for the small and large datasets, respectively. Compared to our proposed system, MelaFind perform similar in the identification of melanoma, but fails in the identification of non-melanoma lesions. The fairest comparison is regarding the results obtained by Stammes et al. that employed a similar annotation scheme to our work, i.e., malignant vs. benign. Our system provided the best specificity results that can be found in the literature, but the sensitivity result for the malignant lesions is lower than other works. We have computed the sensitivity of our approach in classifying melanoma lesions. In the test set, 2 melanoma lesions (P102_C1 and P97_C1) were included in the malignant class. Using the risk threshold of 40%, these two lesions were correctly identified as melanoma; hence, the sensitivity of our proposed approach for melanoma detection is 100%. In any case, the reduced number of HS images in the test set (10 images, 5 benign and 5 malignant) in our study, highly penalizes the results when an HS image is misclassified, especially for the less common class (i.e., melanoma).

## 4. Limitations and Future Directions

Additional research must be carried out to validate and improve the obtained results taking into account the current limitations of this study. One of these limitations is related with the low number of samples in each class (benign: 40 and malignant: 36). Although this number of samples is enough for a preliminary study, our future investigations will target an increase in the number of samples for each class with different types of skins and PSLs to enhance the segmentation and classification results. Moreover, other processing approaches should be investigated, such as developing specific mathematical models for processing the data or the employment of deep learning techniques. Other limitation is related with the low spatial resolution of the HS camera employed in this study. The use of a higher spatial resolution HS camera could improve the results by including spatial features of the PSLs. Another future challenge for this application is the generation of the classification results in real-time while the HS image is captured, providing in-situ diagnosis support. For this task, future research to accelerate the processing framework in specific hardware platforms, such as GPUs (Graphics Processing Unit) or FPGAs (Field-Programmable Gate Array), will be explored. In the future, this system could allow reducing the number of biopsies of non-malignant PSLs, giving more confident to the dermatologist’s diagnosis as well as to facilitate to non-experimented medical doctors (or even patients themselves) the diagnosis of potential malignant lesions.

## 5. Conclusions

The work presented in this paper had the goal of using HSI technology as a non-invasive clinical support system for diagnosing PSLs during dermatological routine practice. A customized HS dermatologic acquisition system for capturing HS data of PSLs was developed, obtaining an HS database composed by 76 images from 61 subjects. Using this HS database, a processing framework to classify the PSLs was proposed and validated using a methodology based on a three data partition fashion (train, validation and test sets), which provides an unbiased evaluation of the final processing model. The proposed framework isolates the PSL pixels in the HS image using a segmentation methodology, and classifies such pixels using a supervised classifier, with the main goal of achieving real-time processing for in-situ diagnosis support.

Two different image segmentation methods were proposed. Both methods combined the K-means and SAM algorithms to identify the PSL pixels using a reference spectral signature library of PSL and normal skin. The first one compared each cluster obtained by the K-means with the library, while the second one compared each pixel from each cluster from the K-means with the library. In addition, different classifiers were employed to obtain the most accurate results in the discrimination of the different types of PSL. The GA algorithm was used to find the optimal hyperparameters for each classifier. The results obtained showed SVM Linear classifier offered better results than the rest of the classifiers, providing an AUC value of 0.89. This preliminary study provides evidence that the combination of HSI and machine learning algorithms allows achieving promising differentiation of PSL types.

## Figures and Tables

**Table 1:** HS Dermatological Labeled Dataset.

Type	#Patients	#Images	#Labeled Pixels
Benign	27	40	7471
Malignant	36	36	8490
Total	618 *	76	15,961

* The total number of patients is not the sum between Benign and Malignant patients due to two patients had several lesion types captured.

**Table 2:** K value using Silhouette, Calinski Harabasz and Davies Bouldin clustering evaluation methods.

*K* Value	Silhouette	Calinski Harabasz	Davies Bouldin
Minimum	2	2	2
Maximum	6	6	7
Most Frequent	2	2	2

**Table 3:** Validation Classification Results.

Classifier	Default Hyperparameters	AUC	Optimized Hyperparameters	AUC
SVM Linear	C=1	0.70	C=94.07	0.89
SVM RBF	C=1 ; γ=1/116	0.66	C=13.41 ; γ=8.43	0.77
SVM Sigmoid	C=1; s=1/116; cf=0	0.50	C=45.75; s=−9.53 ; cf=−14.22	0.83
RF	nTrees=500	0.61	nTrees=3	0.61
ANN	$neurons_{per\;layer}=\left[1\right]$	0.59	$neurons_{per\;layer}=\left[1;3;443;2\right]$	0.61

C: *Cost*; γ: *Gamma*; cf: *Intercept Constant*; s: *Slope*. See Table S2 in the Supplementary Material for more details about the SVM kernel hyperparameters.

**Table 4:** Comparison of the obtained results with the state-of-the-art.

Reference	#Patients	#Images	#Bands	Spectral Range (nm)	Sensitivity (%)	Specificity (%)
Tomatis et al. [12]	1278	1391	15	483–950	80.4 *	75.6
Moncrieff et al. [13]	311	348	8	400–1000	100.0 *^,¥^	5.5
Fink et al. [16]	111	360	10	430–950	100.0 *^,¥^	5.5
Song et al. [17]	55	36	10	430–950	71.4 *^,α^	25.0
Monheit et al. [15]	1257	1612	10	430–950	98.2 *	9.5
Nagaoka et al. [20]	97	134	124	380–780	96.0 *	87.0
Stamnes et al. [21]	-	157	10	365–1000	97.0	97.0
Stamnes et al. [21]	-	712	10	365–1000	99.0	93.0
**Proposed**	61	76	116	450–950	87.5/100.0 *	100.0

* Sensitivity for melanoma detection. ¥ Only reported sensitivity for 3 melanoma lesions. α Only reported sensitivity for 4 melanoma lesions.

## Supplementary Materials

The following are available online at https://www.mdpi.com/2077-0383/9/6/1662/s1, Table S1. HS Dermatological patient stratified assignment labeled dataset. Table S2. Mathematical expressions of the SVM kernels. Table S3. Jaccard coefficient and median values for segmentation validation results for both per centroid and per pixel methods. Figure S1. Average spectral signatures of the labeled PSL (dashed red line) and normal skin (dashed green line) pixels and reference spectral signatures of PSLs (red line) and normal skin (green line). (a) *P13_C1* (malignant PSL). (b) *P14_C1* (benign PSL). (c) *P23_C1* (benign PSL). (d) *P28_C1* (benign PSL). (e) *P69_C1* (benign PSL). (f) *P74_C1* (malignant PSL). (g) *P97_C1* (malignant PSL). (h) *P100_C1* (malignant PSL). (i) *P102_C1* (malignant PSL). (j) *P107_C1* (benign PSL).
