# Supplementary material for: Non-Invasive Skin Cancer Diagnosis Using Hyperspectral Imaging for In-Situ Clinical Support
Source: J Clin Med. 2020 Jun 1;9(6):1662. doi: 10.3390/jcm9061662 (PMC7356572; doi:10.3390/jcm9061662)
Supplement: Supplementary file 1 [file jcm-09-01662-s001.pdf]

Non-invasive Skin Cancer Diagnosis using Hyperspectral Imaging for In-situ Clinical Support
(Supplementary Material)

Table S1. HS dermatological with stratified assignment labeled dataset

Type	#Patients*			#Images			#Labeled Pixels		
	Training	Validation	Test	Training	Validation	Test	Training	Validation	Test
**Benign**	18	4	5	30	5	5	5,254	940	1,277
**Malignant**	26	5	5	26	5	5	6,304	991	1,195
**Total**	**44**	**9**	**10**	**56**	**10**	**10**	**11,558**	**1,931**	**2,472**

*The total number of patients is not the sum between Benign and Malignant patients due to two patients had several lesion types captured

Table S2. Mathematical expressions of the SVM kernels

Kernel	Formula	Hyperparameters
**Linear**	$k(x, y) = x^T \cdot y$	C
**RBF**	$k(x, y) = exp(-\gamma \cdot \|x - y\|^2)$	C, γ
**Sigmoid**	$k(x, y) = tanh(s \cdot x^T \cdot y + cf)$	C, s, cf

Table S2 shows the equations of each kernel and their correspondent hyperparameters to be tuned. The optimization of the linear kernel is based on the adjustment of the cost (C) hyperparameter. This hyperparameter is the constant of constraint violation, which observes if a data sample is classified on the wrong side of the decision limit. In the case of RBF kernel, it is necessary to optimize the cost and the gamma (γ) hyperparameters. Gamma allows adjusting the width of the Gaussian radial basis function. Finally, the sigmoid kernel requires adjusting three parameters: cost, slope (s) and the intercept constant (cf) hyperparameters.

Table S3. Jaccard coefficient and median values for segmentation validation results for both *per centroid* and *per pixel* methods.

Image ID	Per centroid						Per pixel					
	K = 2	K= 3	K = 4	K = 5	K = 6	K = 7	K = 2	K= 3	K = 4	K = 5	K = 6	K = 7
**P15_C1**	0.91	0.92	0.81	0.87	0.61	0.63	0.91	0.92	0.81	0.62	0.85	0.85
**P15_C2**	0.86	0.90	0.74	0.45	0.63	0.63	0.86	0.90	0.75	0.80	0.52	0.65
**P20_C2**	0.78	0.11	0.11	0.09	0.09	0.09	0.14	0.11	0.09	0.09	0.09	0.08
**P60_C1**	0.84	0.71	0.65	0.51	0.51	0.46	0.84	0.65	0.65	0.51	0.51	0.37
**P68_C1**	0.72	0.86	0.82	0.72	0.85	0.85	0.72	0.84	0.85	0.72	0.85	0.85
**P79_C1**	0.81	0.75	0.80	0.80	0.83	0.83	0.81	0.70	0.80	0.80	0.81	0.79
**P96_C1**	0.65	0.79	0.75	0.77	0.77	0.78	0.65	0.80	0.75	0.78	0.78	0.89
**P99_C1**	0.80	0.86	0.83	0.86	0.86	0.82	0.80	0.86	0.84	0.86	0.85	0.85
**P103_C1**	0.84	0.87	0.87	0.87	0.82	0.83	0.84	0.87	0.87	0.87	0.80	0.85
**P113_C1**	0.00	0.00	0.42	0.29	0.26	0.29	0.00	0.00	0.00	0.42	0.21	0.21
**Median**	**0.81**	**0.83**	**0.78**	**0.75**	**0.70**	**0.71**	**0.81**	**0.82**	**0.78**	**0.75**	**0.79**	**0.82**

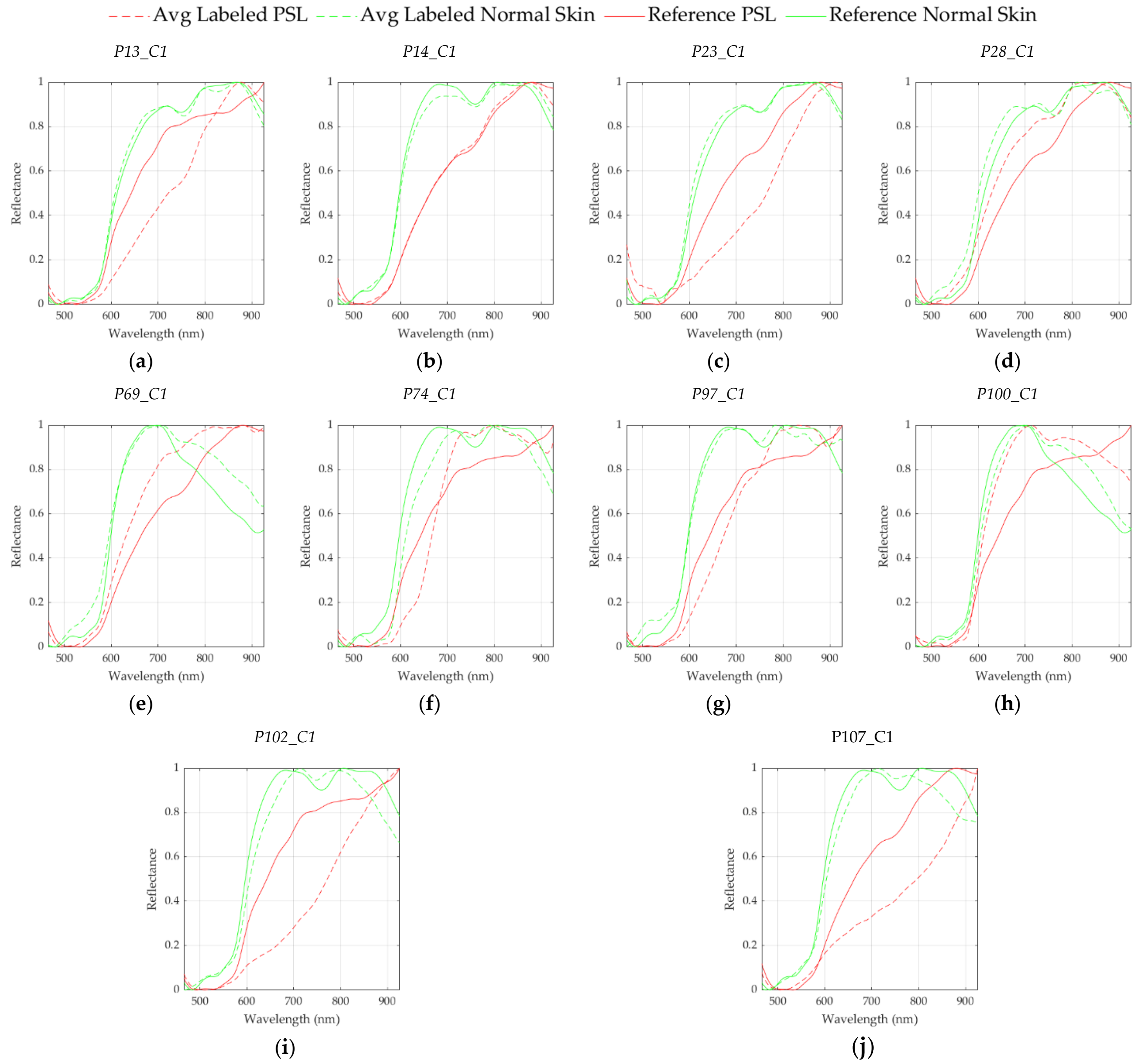

Figure S1. Average spectral signatures of the labeled PSL (dashed red line) and normal skin (dashed green line) pixels and reference spectral signatures of PSLs (red line) and normal skin (green line). (**a**) *P13_C1* (malignant PSL). (**b**) *P14_C1* (benign PSL). (**c**) *P23_C1* (benign PSL). (**d**) *P28_C1* (benign PSL). (**e**) *P69_C1* (benign PSL). (**f**) *P74_C1* (malignant PSL). (**g**) *P97_C1* (malignant PSL). (**h**) *P100_C1* (malignant PSL). (**i**) *P102_C1* (malignant PSL). (**j**) *P107_C1* (benign PSL).
